# TRPC3 overexpression promotes angiotensin II-induced cardiac dysfunction

**DOI:** 10.1186/2050-6511-13-S1-A83

**Published:** 2012-09-17

**Authors:** Bernhard Doleschal, Stefan Wolf, Marie-Sophie Huber, Gerald Wölkart, Gudrun Antoons, Klaus Groschner

**Affiliations:** 1 Department of Pharmacology and Toxicology, Institute of Pharmaceutical Sciences, University of Graz, 8010 Graz, Austria; 2 Division of Cardiology, Medical University of Graz, 8036 Graz, Austria; 3 Institute of Biophysics, Medical University of Graz, 8010 Graz, Austria

## Background

TRPC3 was recently demonstrated as a player in pathogenesis of cardiac hypertrophy, while the potential pro-arrhythmogenic role of TRPC3 is incompletely understood. Using a TRPC3 transgenic overexpression mouse model, we examined the involvement of TRPC3 in cardiac actions of angiotensin II (AngII).

## Methods

AngII effects on cardiac functions were characterized in Langendorff perfused hearts. Single ventricular myocytes were isolated and field-stimulated to measure effects on sarcomere shortening and Ca^2+^ transients. Furthermore, L-type Ca^2+^ channel current, action potentials and non-selective ion currents were analyzed electrophysiologically.

## Results

AngII (100 nM) reduced left ventricular pressure (LVP) within 2 min to 64%, +dP/dt to 50% and −dP/dt to 55% of control in TRPC3(+/−) hearts, while even producing a positive inotropic effect in wild-type (WT) hearts. Simultaneously, ECG recordings demonstrated AngII-induced episodes of acute arrhythmogenicity in all TRPC3(+/−) hearts (n = 6), whereas rhythm of WT hearts (n = 6) remained unaffected. The AngII-induced impairment of cardiac functions in TRPC3(+/−) hearts was partially reversed by Pyr3 (30 µM). The amplitude of Ca^2+^ transient was significantly higher (p < 0.05; n = 60) in myocytes from TRPC3(+/−) mice ([Ca^2+^] F/F_0_ 0.354 ± 0.024) as compared to WT ([Ca^2+^] F/F_0_ 0.262 ± 0.021). Also, the time constant (τ) of Ca^2+^ decline was different between WT (0.196 ± 0.009 ms; n = 61) and TRPC3(+/−) (0.170 ± 0.008; n = 67; p < 0.05). Sarcomere shortening showed no significant difference between the two groups (3.80 ± 0.69% vs. 3.52 ± 0.65%; n = 10) whereas the SR-loading estimated from rapid application of caffeine (20 mM) revealed an increased SR loading of up to 40% in TRPC3(+/−) myocytes as compared to WT (p < 0.05; n = 43). The time constant of Ca^2+^ decline during caffeine challenge was also significantly changed (p < 0.05) in TRPC3(+/−) myocytes (3.04 ± 0.44 ms; n = 11) as compared to WT cells (1.65 ± 0.158 ms; n = 16). Importantly, AngII (100 nM) induced a rise in diastolic Ca^2+^ levels, which was accompanied by irregular contractions in TRPC3(+/−) but not in WT myocytes. The rise in the diastolic Ca^2+^ levels was significantly suppressed by Pyr3 (10 µM; n = 16), SEA 0400 (1 µM; n = 14) and KN-93 (1 µM; n = 12). Electrophysiological characterization of L-type voltage-gated Ca^2+^ currents and action potentials revealed that baseline electrophysiological parameters were not affected by TRPC3 overexpression, while AngII induced a transient prolongation of action potential duration only in TRPC3(+/−) myocytes. This TRPC3-dependent response was associated with a higher incidence of delayed afterdepolarizations.

## Conclusions

Our results demonstrate that AngII modulation of cardiac functions is strictly dependent on TRPC3 expression and suggest a key role of TRPC channels in AngII-mediated arrhythmogenicity.

